# Intrinsic Connectivity Networks in the Self- and Other-Referential Processing

**DOI:** 10.3389/fnhum.2020.579703

**Published:** 2020-11-10

**Authors:** Gennady G. Knyazev, Alexander N. Savostyanov, Andrey V. Bocharov, Evgeny A. Levin, Pavel D. Rudych

**Affiliations:** ^1^Laboratory of Psychophysiology of Individual Differences, Institute of Physiology and Basic Medicine, Novosibirsk, Russia; ^2^Joint Laboratory of Psychological Genetics at the Institute of Cytology and Genetics SB RAS, Institute of Physiology and Basic Medicine, Novosibirsk, Russia; ^3^E.N. Meshalkin National Medical Research Center, Novosibirsk, Russia

**Keywords:** default mode network, central executive network, salience network, self-referential processing, trait adjective judgment task, fMRI

## Abstract

Neuroimaging studies have revealed a multitude of brain regions associated with self- and other-referential processing, but the question how the distinction between self, close other, and distant other is processed in the brain still remains unanswered. The default mode network (DMN) is the primary network associated with the processing of self, whereas task-positive networks (TPN) are indispensable for the processing of external objects. We hypothesize that self- and close-other-processing would engage DMN more than TPN, whereas distant-other-processing would engage TPN to a greater extent. To test this hypothesis, we used functional magnetic resonance imaging (fMRI) functional connectivity data obtained in the course of a trait adjective judgment task while subjects evaluated themselves, the best friend, a neutral stranger, and an unpleasant person. A positive association between the degree of self-relatedness and the degree of DMN dominance was revealed in cortical midline structures (CMS) and the left lateral prefrontal cortex. Relative to TPN, DMN showed greater connectivity in me than in friend, in friend than in stranger, and in stranger than in unpleasant conditions. These results show that the less the evaluated person is perceived as self-related, the more the balance of activity in the brain shifts from the DMN to the TPN.

## Introduction

The nature of self is one of the most controversial questions throughout the history of philosophy and science. Different approaches emphasize different aspects of this construct including emotional (Fossati et al., 2003), cognitive (Turk et al., 2003), and social (Frith and Frith, 2003) self. Moreover, some authors deny its reality altogether claiming that the self is just an illusion of our perception (e.g., Hood, 2012). Despite these controversies, neuroscientific evidence reliably shows a set of brain regions, which are robustly associated with self-referential processing across functional domains (Northoff et al., 2006). In particular, cortical midline structures (CMS) including different parts of the medial prefrontal cortex (MPFC), the anterior cingulate cortex (ACC), the medial parietal cortex, the posterior cingulate cortex (PCC), and the retrosplenial cortex have been proposed as the system underlying the human self (Northoff and Bermpohl, 2004; Qin and Northoff, 2011). However, many studies show that self-processing is not limited to the CMS and includes many other brain regions, such as ventro- and dorsolateral prefrontal cortex (DLPFC), temporal regions, temporoparietal cortex, insula, and a number of subcortical regions (Gazzaniga, 1998; Kircher et al., 2000; Damasio, 2003; Gallagher and Frith, 2003; Vogeley and Fink, 2003; Gillihan and Farah, 2005; Northoff et al., 2006; Morin and Michaud, 2007; Vanderwal et al., 2008).

Whether these regions are specifically involved in self-processing or participate in a broader range of social cognitive tasks is not a simple question. One of the most popular approaches to the study of self-referential vs. other-referential processing is the trait adjective judgment task. In this task, participants are asked to judge whether trait adjectives properly describe the participants themselves or some other person. Neuroimaging studies using this paradigm have reported selective recruitment of a region in the MPFC when making judgments about the self, relative to others (Kelley et al., 2002; Heatherton et al., 2006). Meta-analyses of functional magnetic resonance imaging (fMRI) studies showed two areas within the MPFC, namely the ventral and dorsal MPFC, that are most important in distinguishing self from others. Specifically, the ventral MPFC is responsible for processing information relevant for self, whereas the dorsal MPFC is responsible for other-referential processing (van der Meer et al., 2010; Denny et al., 2012). Ventral MPFC activation was also observed during comparisons between the self and similar others (Moore et al., 2014). However, some authors argue that dorsal and ventral MPFC do not actually distinguish between self and others, but rather differentiate whether an action is relevant or irrelevant to the task at hand (Nicolle et al., 2012; Cook, 2014). It has also been suggested based on meta-analyses of fMRI studies that whereas the ventral MPFC is responsible for processing information relevant for self, posterior midline structures, such as the PCC and the adjacent cuneus might preferentially process information related to others (Qin and Northoff, 2011; Denny et al., 2012). Pfeifer et al. (2007) showed that a relative involvement of MPFC and posterior midline structures during self- and other-processing changes in the course of development.

Most of the findings discussed so far relate to the study of regional activation effects. Functional connectivity studies bring out conceptually different kind of evidence. A surge of connectivity research is associated with the discovery of the so-called resting state networks, i.e., spatially separate, but functionally related regions, which exhibit temporal correlation even in the absence of a task (Biswal et al., 1995). Later studies showed that these networks are highly functionally relevant and could also be recovered from fMRI data obtained in different kinds of tasks (Smith et al., 2009). Therefore, the term intrinsic connectivity networks (ICNs) is a more appropriate label for their designation (Seeley et al., 2007). Perhaps the most intriguing theme in the study of ICNs is the contradistinction of the so-called task-positive (TPN) and task-negative (TNN) networks. The default mode network (DMN), which includes areas in dorsal and ventral medial prefrontal cortices, medial and lateral parietal cortex, and parts of the medial and lateral temporal cortices, has been labeled TNN because it decreases its activity in most kinds of tasks that require attention to the environment, in contrast with most other ICNs, which show activity increases in such kind of tasks (Fox et al., 2005). Perhaps most important of these latter networks are the frontoparietal executive control network (CEN), which is anchored in the DLPFC and the salience network (SAL) anchored in the anterior insula (Dosenbach et al., 2007; Seeley et al., 2007; Vincent et al., 2008). These networks are involved in sustaining attention in the course of a task and directing it to most salient stimuli, respectively. The TNN and the TPN are supposed to correlate negatively with each other (Fox et al., 2005) and an appropriate balance between their activities is presumably a prerequisite of mental health and adequate brain functioning (Hamilton et al., 2011, 2013; Menon, 2011). Although the existence of “anticorrelation” between the TPN and the TNN has been called into question based on the fact that it could be artificially introduced by global signal regression used for data cleaning (Murphy et al., 2009), later studies showed that it could be observed even without global signal regression (e.g., Allen et al., 2012; Chai et al., 2012). Nevertheless, the accumulated evidence shows that the TNN label is imprecise and might even “transmit a profound misconception about the functional role of the default network in cognition” (Spreng, 2012, p. 1), because DMN is not strictly speaking “task negative” and is frequently engaged in goal-directed cognition. Correspondingly, although the DMN and the TPN may show anticorrelation in a resting state, they may cooperate with each other in some experimental tasks (e.g., Beaty et al., 2016). Summing up, rather than emphasizing “task negativeness” or “task positiveness” in general, the distinction between the DMN on the one hand and the CEN and SAL on the other should be considered in terms of the cognitive processes they are involved in. This, however, also does not bring out a clear-cut picture, particularly in the context of self- and other-referential processing. DMN is the primary network associated with internally oriented attention and self-referential processing (Raichle et al., 2001; Buckner et al., 2008; Davey et al., 2016), but it may be involved in a broader range of social (Lombardo et al., 2009; Mars et al., 2012; Li et al., 2014; Laurita et al., 2017) and other (e.g., Li et al., 2012; Beaty et al., 2016; Vatansever et al., 2018) cognitive processes as well. CEN and SAL are mostly associated with externally oriented attention (e.g., Duncan, 2013; Di and Biswal, 2014; Hugdahl et al., 2015), but parts of these networks could be involved in explicit self-referential processes (Whitfield-Gabrieli et al., 2011; Molnar-Szakacs and Uddin, 2013; Davey et al., 2016). Connectivity studies of fMRI data acquired during self- vs. other-referential tasks show importance of information flow from some regions outside the DMN, such as the left inferior frontal gyrus and caudate nuclei, to different hubs of the DMN (Grigg and Grady, 2010; Soch et al., 2017).

Although these recent findings show that the distinction between the DMN and the TPN is not so straightforward as was initially suggested, the importance of this distinction for the understanding of many normal and pathological processes raises no doubts (e.g., Hamilton et al., 2011, 2013; Menon, 2011; Figueroa et al., 2015; Norman et al., 2017; Querne et al., 2017). Much evidence shows that a “dominance” of the DMN over the TPN is observed in pathological conditions, such as depression (Hamilton et al., 2011, 2013; Knyazev et al., 2016, 2018), social anxiety (Liao et al., 2010), and PTSD (Daniels et al., 2010), and this is interpreted as a consequence of enhanced self-focus and diminished attention to the environment (Hamilton et al., 2011; Menon, 2011). On the other hand, meditation practices targeted at diminishing self-focus and maintaining attention to the immediate experience have been shown to decrease DMN and increase TPN activity and connectivity (Brewer et al., 2011; Kemmer et al., 2015; Taren et al., 2017). It could be expected that differential involvement of the DMN and the TPN should show up in the context of self- and other-referential processing as well.

The terms “dominance” and “balance” imply a sort of competition between different networks and different cognitive processes. The notion of such competition is a useful and frequently used model in cognitive science (e.g., the horse-race model of go and stop process, Logan et al., 2014, or the model of interhemispheric competition, Zaidel et al., 1990). The triple network model suggested by Menon emphasizes that the DMN, CEN, and SAL are involved in extremely wide range of cognitive tasks, and their responses are often antagonistic. “The CEN and SN typically show increases in activation during stimulus-driven cognitive and affective information processing, whereas the DMN shows decreases in activation during tasks in which self-referential and stimulus-independent memory recall is not crucial” (Menon, 2011, p. 500). Due to this apparent antagonism between the DMN and the TPN, the notion of competition could be meaningfully applied in this case also and could be operationalized in terms of “balance” and “dominance.” In the context of activity, the DMN-TPN balance could be evaluated by revealing the degree of activation in DMN and/or TPN brain regions. In terms of connectivity, this actually boils down to revealing cortical areas that are stronger connected to DMN than TPN and vice versa. Given that all three networks may be involved in all underlying processes, measuring the balance of their activity/connectivity is more important than the study of each one of these networks separately. In normal conditions, the DMN-TPN balance should be dynamic and state-dependent. Long-lasting dominance of one network over the other could be observed either in pathological condition, or as a result of special training.

It is important to note that different others have different degrees of closeness to the self. The overlap between self and other representation has repeatedly been noted (e.g., Cross et al., 2002; Lombardo et al., 2009; Laurita et al., 2019), and the degree of this overlap may be modeled by a single dimension spanning from self-centeredness on the one pole to self-other connectedness on the other (Trautwein et al., 2014). In terms of brain activity, recent studies show that at least in some cultural contexts, self-referential and close-other-referential processing could be indistinguishable, whereas they are always distinguishable for self-referential and distant-other-referential processing (Zhang et al., 2006; Zhu et al., 2007; Zhu and Han, 2008). It could be suggested that self- and close-other-referential processing may engage the DMN more than the TPN, whereas distant-other-referential processing may engage the TPN more than the DMN. To the best of our knowledge, a comparison of relative involvement of DMN and TPN (in terms of either activation or connectivity measures) in the processing of self and different others varying in the degree of their closeness to the self is lacking in the literature. The degree of subjectively experienced closeness to the self may not so much depend on familiarity with a person as on subjective evaluation of his/her similarity to the self. Because most healthy people tend to evaluate themselves positively rather than negatively (Baumeister, 1999), the degree of subjectively experienced similarity of a person to the self might indirectly be evaluated based on the degree of approval/disapproval of this person. It could be expected that the best friend or close relative should receive the highest approval and would be perceived the closest to the self, whereas an unpleasant person should receive the lowest approval and would be perceived as most distant from the self. A neutral stranger may fall in between.

In this study, using fMRI functional connectivity data, we aimed to directly compare DMN vs. TPN connectivity during self- and other-appraisal in the trait adjective judgment task for different others varying in the degree of their closeness to the self (i.e., close friend, stranger, and an unpleasant person). To this end, we used the data, which have already been described in our previous article (Knyazev et al., 2020). In this article, we were specifically interested in the effect of cultural values, as measured by self-reported independent and interdependent self-construal, on DMN connectivity during self- and other-referential processing. We showed that individualist values predispose to a greater DMN engagement during self-processing, whereas collectivist values predispose to its greater engagement in other-processing. We did not analyze, however, the TPN connectivity and the question of DMN-TPN balance remained unsolved. Here, we hypothesized that the DMN vs. TPN balance should linearly decrease during evaluation of self, close-other, distant-other, and an unpleasant person.

## Materials and Methods

### Participants

Fifty participants were initially enrolled. Most participants were undergraduate and postgraduate students and staff members of Novosibirsk State University. Three participants were later excluded from the analysis due to excessive fMRI artifacts, thus leaving 47 participants (26 females; mean age, 23.5 years; SD, 4.9). Exclusion criteria were major medical illness, history of seizures or substance abuse or dependance, as well as all contraindications against MRI. The study conforms with World Medical Association Declaration of Helsinki and was approved by the Institute of Physiology and Basic Medicine ethical committee. All participants gave written informed consent.

### Stimuli and Task

For the trait adjective judgment task, a list of 150 adjectives was generated. Most words were taken from personality questionnaires, others from descriptions of appearance. Thirty-five experts (lecturers and students from humanitarian department of Novosibirsk State University) rated each adjective using five-point Likert scale on emotional valence and emotional arousal. Based on the average rating, 30 positive, 30 neutral, and 30 negative adjectives were selected so that they did not on average differ on length and the number of vowels.

In the beginning (outside the scanner), participants were asked to choose three persons. The identities of these persons they did not have to reveal to the experimenter. They had to choose the best friend or close relative with whom they currently had most intimate and confidential relationships (hereafter “Friend”), a neutral stranger, whom they knew superficially and with whom they had no personal relationships (hereafter “Stranger”), and an unpleasant person, whom they disliked or with whom they were in a bad relationship (hereafter “Unpleasant”). After that, participants were asked to rate the selected persons on emotional valence scale ranging from −5 to +5. Next, the upcoming trait adjective judgment task was explained to them and they were presented with a training session, in which they had to rate a randomly chosen target (i.e., “Me,” “Friend,” “Stranger,” or “Unpleasant”). Next, within the scanner, the procedure consisted of the same four conditions, which in different subjects alternated pseudo-randomly. In the beginning of each condition, a cue appeared on the screen (e.g., “You” or “Friend”) and it remained at the screen throughout the condition. First, the participant was asked to think for 1 min about this person and to recall his/her characteristics. In the subsequent task, which later was used for the analysis, subjects were presented with adjectives and were asked to judge whether the respective trait applied to the evaluated person. In the beginning of each trial, the pause between the upcoming fMRI frame onset and adjective presentation onset was randomly varied between 100- and 2,350-ms intervals. Participants responded by pressing the left (No) or right (Yes) button using the index fingers of their left and right hand and the adjective instantly disappeared. The next trial started 5 s after the onset of adjective presentation. Therefore, each condition, which in the data analysis was treated as a block (see below), lasted for 90 * 5 = 450 s and the 90 adjectives were balanced by valence per each condition. Word order within the condition was randomized, and no adjective was presented twice.

### fMRI Data Acquisition

Whole brain fMRI data were acquired with an EPI sequence on a 3.0-Tesla scanner Philips Ingenia 7FN8GDI 3.0 T. The first five volumes in the beginning of each session were discarded to allow for scanner equilibration effects, leaving 225 volumes for each of the four sessions (TR = 2.5 s; TE = 35 ms; flip angle = 90°; percent phase FOV = 100; 96 × 94 matrix, 25 slices of 5 mm thickness, no gap). High-resolution 1-mm T1-weighted structural scans were acquired with a 3D MP-GR sequence (TR = 7.8 ms, TE = 3.76 ms, 252 × 227 matrix).

### General Linear Model Analysis of Task-Related Blood Oxygen Level-Dependent Activation

Such kind of data could be analyzed using an event-related design, where presentation of each adjective would be treated as an event. This would entail considering an additional factor, namely, the adjective valence. Although the question of how the valence factor interacts with the target factor is in itself interesting, it was not the question that interested us in this study. Besides, the analysis of event-related dynamic functional connectivity is not so far firmly established for relatively short events, as is the case here. Therefore, in this study, the analysis of both task-related BOLD activation and task-related functional connectivity, was performed using a block design. To account for the effect of negative, neutral, or positive emotional valence, we used parametric modulators (−1, 0, or 1, respectively) along with parametric modulators describing the subject’s response (−1 or 1). However, for the sake of completeness and for BOLD activation only, we additionally performed event-related analysis of the effect of valence factor and its interaction with the target factor. The analysis of task-related BOLD activation was performed using the SPM-12 toolbox. Preprocessing included slice-time correction, realignment using rigid body transformation, co-registration and normalization to the Montreal Neurological Institute (MNI) template, resampling to 2 × 2 × 2 mm, and smoothing (full-width half-maximum, 6 mm). We checked for motion parameters, which might induce false-positive results (Van Dijk et al., 2012). The cutoff for motion quality of the images was set at 2 mm for the three translation planes, and all participants who exceeded this motion threshold were excluded from the subsequent analysis. Next, for each subject, a general linear model (GLM) was set up by specifying the onsets and durations of the four task conditions as boxcar functions with on and off points corresponding to the start and the end of each block. Single subjects’ hemodynamic response was modeled by convolving the boxcar functions with a canonical hemodynamic response function (HRF).

In the study of both the DMN and the TPN, an important question is whether the observed effect depends solely on the content of a task, or is confounded by task difficulty, because in attention tasks, DMN tends to decrease its activity proportionally to task demands (Greicius et al., 2003). Reaction time (RT) is frequently used as a proxy for task difficulty. We therefore included RT as additional parametric modulator in the design matrix of both BOLD activation and connectivity analyses. Therefore, the design matrix in the first-level analysis included 15 regressors. First, adjective presentation was modelled by a stick function indicating the onset of each trial, which was convolved with the canonical HRF. The stick function was modulated by three parametric modulators (adjective valence, response, and RT). Next, the four regressors for task blocks (Me, Friend, Stranger, and Unpleasant), six realignment parameters, and one constant were followed. Data were high-pass filtered with a cutoff at 128 s, and an autoregression model of polynomial order 1 was used to account for temporally correlated residuals. Model estimation was performed using a restricted maximum likelihood (ReML) fit. After model estimation, contrast images representing the effects of each experimental condition were computed for each participant and submitted to a second-level random-effects analysis. Modeling the effect of the degree of target’s closeness to the subject’s self on neuronal activation was performed using a *t*-contrast and attributing to the four targets linearly decreasing weights, i.e., Me = 3, Friend = 1, Stranger = −1, and Unpleasant = −3. Such weighting models a linear decrease from Me to Unpleasant. However, using the weights (2, 1, −1, −2) produced the same results. Next, in order to visualize the degree of activation in significant clusters in each condition, an F contrast was specified using the identity matrix [i.e., in MATLAB eye (4)], which allowed us to reveal all effects of interest in the four experimental tasks. Participant’s age and sex were entered as second-level covariates of no interest. Voxel height threshold was set at *p* < 0.001 and a family-wise error (FWE)-corrected cluster threshold at *p* < 0.05.

Finally, although the effect of valence factor and its interaction with the target factor were not of principal interest in this study, we nevertheless performed such analysis for the sake of completeness. Because adjectives of different valences were presented randomly within each condition, such analysis was only possible using event-related design. Therefore, in this case, presentation of each adjective was modeled by a boxcar function with on and off points corresponding to the time at which the adjective was presented and the time when the subject pressed the response button, respectively. This function was convolved with the canonical HRF, and the GLM design matrix for each subject included separate regressors for negative, neutral, and positive adjectives within each of the four conditions, six realignment parameters, and one constant. After model estimation, contrast images representing the effects of each adjective valence within each experimental condition were computed for each participant and submitted to a second-level random-effects analysis.

### Functional Connectivity Analysis

Task-related functional connectivity was analyzed using a block design as implemented in the CONN fMRI functional connectivity toolbox (v17.f^1^) and is described in detail in Whitfield-Gabrieli and Nieto-Castanon (2012). After slice-time correction, realignment, co-registration, normalization, resampling, and smoothing, data denoising was performed by regressing out confounding effects related to white matter (WM)/cerebrospinal fluid (CSF) signal (characterized by three dimensions each, representing the variability of BOLD signal time series observed within those areas), as well as motion parameters (six dimensions with first-order derivative) using the CompCor method (Behzadi et al., 2007) for identifying principal components associated with segmented WM and CSF implemented in the CONN toolbox (Whitfield-Gabrieli and Nieto-Castanon, 2012). Besides, the main effect of task and its first temporal derivative as well as artifactual time points were included in first-level covariates. Adding regressors accounting for task effects prevents the main effect of task to drive the estimation of correlations in functional connectivity analysis (Whitfield-Gabrieli and Nieto-Castanon, 2012). The option “weighted GLM” offered in the CONN toolbox allows to describe each task condition by a boxcar function which is convolved with a canonical HRF. As has been described above for the first-level analysis of BOLD activation, the three parametric modulators (adjective valence, response, and RT) were also included in the first-level design matrix.

There are different strategies for defining regions of interest (ROI) or seeds in functional connectivity analysis. They could be identified as peak voxels in the GLM analysis of BOLD activation in a task or they could be found *via* independent component analysis. Last, *a priori* ROIs could be defined by the MNI coordinates derived from published fMRI studies. In this study, we aimed to investigate the well-known ICNs described in previous fMRI studies. Therefore, the last method was used as the most straightforward strategy (see e.g., Crittenden et al., 2016; Letzen et al., 2016; Vervoort et al., 2016 for a similar approach). MNI coordinates of DMN and TPN seeds were taken from the CONN database. Specifically, four seeds were selected to represent the DMN: MPFC (1, 55, −3), PCC (1, −61, 38), and left (−39, −77, 33) and right (47, −67, 29) lateral parietal cortex (Fox et al., 2005). The TPN was also represented by four seeds, which included left (−43, 33, 28) and right (41, 38, 30) DLPFC and left (−44, 13, 1) and right (47, 14, 0) anterior insula (Dosenbach et al., 2007).

Maps of Fisher-transformed bivariate correlations between the seed ROI timecourse and all other voxels were used in the second-level GLM analyses. The factorial design included two within-subject factors—task (self- and other-referential tasks) and network (DMN and TPN). T-contrast was used to model a linear relationship between the degree of self-closeness (i.e., Me = 3, Friend = 1, Stranger = −1, and Unpleasant person = −3) and the degree of DMN dominance [DMN > TPN, contrast weights: (1, 1, 1, 1, −1, −1, −1, −1), for the four DMN and the four TPN seeds, respectively]. Participant’s age and sex were entered as second-level covariates of no interest. False-positive control was implemented through a combination of voxel-level height threshold *p* < 0.001 and cluster-level extent threshold, FWE-corrected cluster-level *p* < 0.05). We used nonparametric testing (5,000 permutations) implemented in the CONN toolbox.

## Results

### Behavioral Data

Average (SD) ratings of selected persons on emotional valence scale were as follows: Friend, 4.43 (0.68); Stranger, 0.98 (0.99); and Unpleasant, −3.34 (1.22). Pairwise comparisons using paired-sample *t*-test showed that all three differences were highly significant (all *p* < 0.001). Repeated measures ANOVA with task (Me/Friend/Stranger/Unpleasant) and adjective valence (negative/neutral/positive) as factors and the number of choices as the outcome showed significant main effects of task (*F*_(3,138)_ = 16.0, *p* < 0.001) and valence (*F*_(2,92)_ = 238.4, *p* < 0.001), and a significant interaction between the factors (*F*_(6,276)_ = 95.9, *p* < 0.001). The main effect of task showed that affirmative choices were on average more frequently made in the Me and Friend than in the Stranger and Unpleasant person conditions. The main effect of valence showed that, on average, positive descriptions were endorsed most frequently, whereas negative ones were endorsed least frequently. The interaction effect indicated that negative characteristics were more frequently selected for the description of Unpleasant person and positive ones in all other cases. Mean (SD) scores of the four targets in the trait adjective judgment task were calculated as a sum of ratings of all endorsed adjectives: Me, 51.1 (13.5); Friend, 63.1 (11.8); Stranger, 42.7 (18.6); and Unpleasant, 31.2 (18.5). Pairwise comparisons using paired-sample *t*-test showed that all differences were significant. The same analysis was performed using RT as the outcome. Only the main effect of task was significant (*F*_(3,138)_ = 7.7, *p* < 0.001). On average, participants took less time for decision-making while evaluating themselves and Friend than while evaluating Stranger and Unpleasant person. Mean (SD) RTs (in ms) were as follows: 1,400 (604), 1,291 (678), 1,543 (750), and 1,650 (850) for Me, Friend, Stranger, and Unpleasant person, respectively. Because the passage of time and the repeated presentation of the same adjectives might have influenced the behavior, the same repeated measures ANOVAs were performed with block order regardless of task (four levels) and adjective valence (negative/neutral/positive) as factors and the number of choices and RT as outcomes. In both cases, neither the main effect of block order, nor its interaction with adjective valence was significant (all *p* > 0.1). As an additional test of the effect of repeated presentation, we calculated the mean endorsement across subjects of each adjective in each block regardless of task. Repeated measures ANOVA was then performed, which treated the adjectives as cases and the four blocks as repeated measures. This analysis also did not yield a significant effect of time (*p* > 0.9).

### GLM Analysis of BOLD Activation

**Figure 1:**
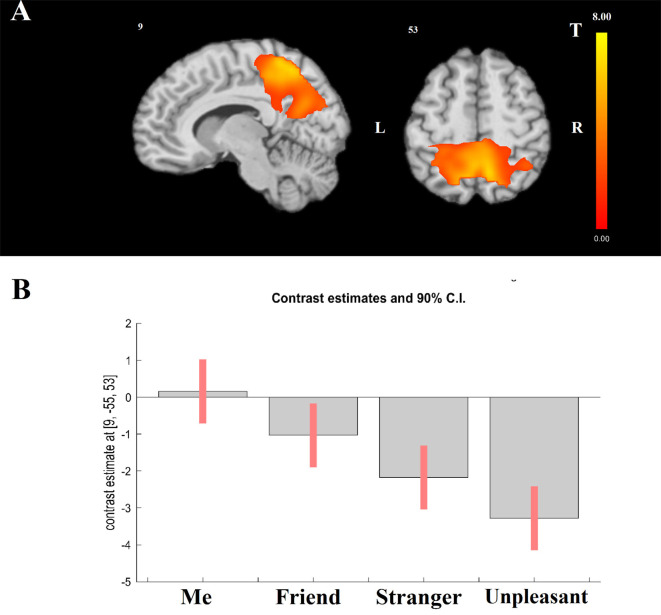
General linear model (GLM) results. Effect of modeling a linear relationship between self-closeness and BOLD activation using a contrast: Me = 3, Friend = 1, Stranger = −1, Unpleasant = −3. **(A)** The localization of significant cluster. **(B)** F Contrast for the effects of interest. Contrast estimates and 90% confidence intervals for BOLD activation in the four experimental tasks within the center of the significant cluster presented at panel **(A)**.

Modeling a linear relationship between the degree of self-closeness and brain activation (i.e., Me = 3, Friend = 1, Stranger = −1, and Unpleasant person = −3) yielded a significant effect in parietal cortical regions (*x* = 9, *y* = −55, *z* = 53; *T*_(1,322)_ = 7.04, cluster-level FWE-corrected *p* < 0.001; Figure 1A). The opposite effect was not significant. As Figure 1B shows, activation in the center of the significant cluster is higher in Me than in Friend, in Friend than in Stranger, and in Stranger than in Unpleasant person condition.

**Figure 2:**
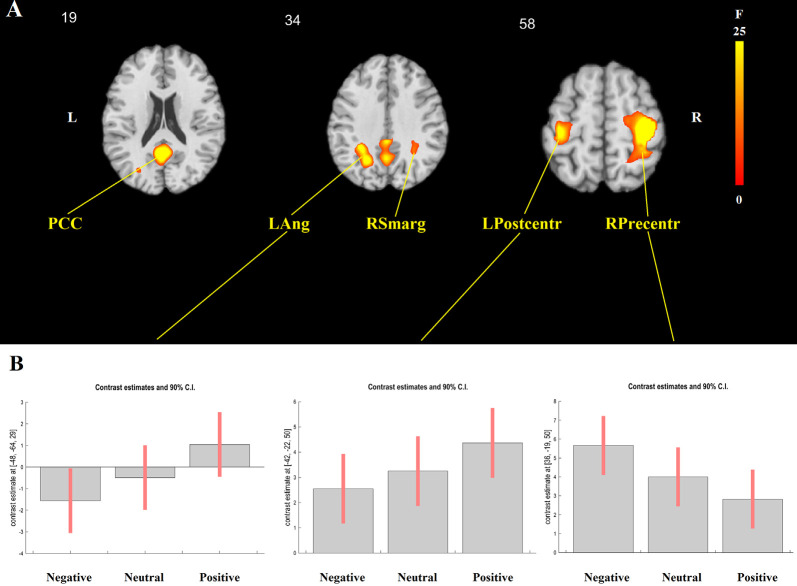
GLM results. The main effect of adjective valence. **(A)** The localization of significant clusters. **(B)** *F* Contrast for the effects of interest. From left to right: contrast estimates and 90% confidence intervals for BOLD activation during the presentation of negative, neutral, and positive adjectives in the left angular gyrus (LAng), the left postcentral gyrus (LPostcentr), and the right precentral gyrus (RPrecentr). In the posterior cingulate cortex (PCC) and the right supramarginal gyrus (RSmarg), the pattern was similar to the one shown for the left angular gyrus.

The event-related analysis of the effect of target and adjective valence on brain activation was additionally performed. The main effect of target was significant in the right superior parietal lobule (*x* = 27, *y* = −64, *z* = 50; *F*_(3,552)_ = 7.87, cluster-level FWE-corrected *p* = 0.024), where activation was highest in Me and lowest in Unpleasant person condition. The main effect of adjective valence was significant in five clusters, including the PCC (*x* = −3, *y* = −55, *z* = 20; *F*_(2,552)_ = 14.88, cluster-level FWE-corrected *p* = 0.002), the left angular gyrus (*x* = −48, *y* = −64, *z* = 29; *F*_(2,552)_ = 25.22, cluster-level FWE-corrected *p* = 0.047), the right supramarginal gyrus (*x* = 51, *y* = −61, *z* = 32; *F*_(2,552)_ = 21.41, cluster-level FWE-corrected *p* = 0.049), the right precentral gyrus (*x* = 36, *y* = −19, *z* = 50; *F*_(2,552)_ = 28.99, cluster-level FWE-corrected *p* < 0.001), and the left postcentral gyrus (*x* = −42, *y* = −22, *z* = 50; *F*_(2,552)_ = 14.33, cluster-level FWE-corrected *p* = 0.013; Figure 2A). In the PCC, left angular, right supramarginal, and left postcentral gyri, the activation was highest for positive and lowest for negative adjectives, whereas the opposite pattern was observed in the right precentral gyrus (Figure 2B).

**Figure 3:**
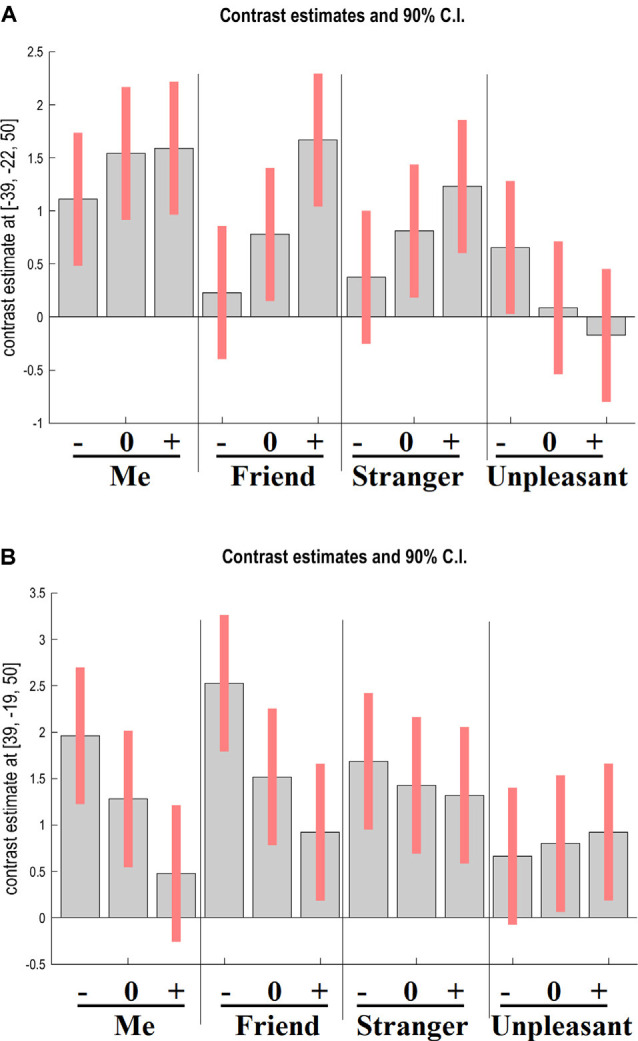
GLM results. The interaction between target and adjective valence. Contrast estimates and 90% confidence intervals for BOLD activation in the left postcentral gyrus **(A)** and the right precentral gyrus **(B)** during the four experimental tasks upon presentation of negative (−), neutral (0), and positive (+) adjectives. Note that activation of the left sensorimotor area reflects the tendency to respond “Yes” (i.e., to press the right-hand button), whereas activation of the right sensorimotor area reflects the tendency to respond “No” (i.e., to press the left-hand button).

The interaction between target and adjective valence factors was significant in the right precentral gyrus (*x* = 39, *y* = −19, *z* = 50; *F*_(6,552)_ = 9.17, cluster-level FWE-corrected *p* = 0.003) and the left postcentral gyrus (*x* = −39, *y* = −22, *z* = 50; *F*_(6,552)_ = 13.72, cluster-level FWE-corrected *p* < 0.001). As Figure 3 shows, activation in the left postcentral gyrus increased with adjective valence for Me, Friend, and Stranger and decreased for Unpleasant person. The opposite dynamic was observed in the right precentral gyrus.

### Analysis of Functional Connectivity

**Figure 4:**
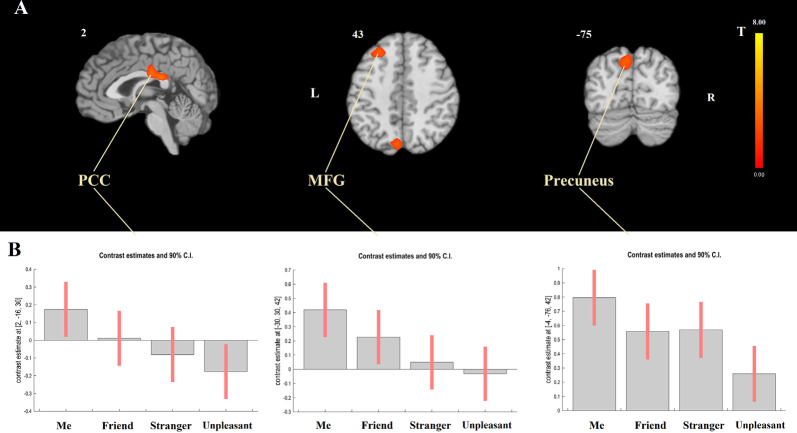
Functional connectivity results. **(A)** Effect of modeling a linear relationship between the degree of self-closeness and the degree of default mode network (DMN) dominance using a contrast: me = 3, friend = 1, stranger = − 1, unpleasant = −3 and DMN > task-positive networks (TPN; 1, 1, 1, 1, −1, −1, −1, −1). **(B)** *F* Contrast for the effects of interest. The figure shows contrast estimates and 90% confidence intervals for the four experimental tasks in the center of the three significant clusters presented at panel **(A)**; [PCC: 2, −16, 30; middle frontal gyrus (MFG): −30, 30, 42, and precuneus: −4, −76, 42]. PCC, posterior cingulate cortex; MFG, middle frontal gyrus.

Modeling a linear relationship between the degree of self-closeness (i.e., Me = 3, Friend = 1, Stranger = −1, and Unpleasant person = −3) and the degree of DMN dominance [DMN > TPN, contrast weights: (1, 1, 1, 1, −1, −1, −1, −1), for the four DMN and the four TPN seeds, respectively] yielded three significant clusters, two of which were situated in posterior CMS regions (PCC and precuneus) and one in the left middle frontal gyrus (MFG; Table 1; Figure 4A). The opposite effect was not significant. As Figure 4B shows, for PCC and MFG, the degree of DMN dominance (i.e., stronger connectivity with DMN than with TPN seeds) is higher in Me than in Friend, in Friend than in Stranger, and in Stranger than in Unpleasant condition. For precuneus, it is higher in Me than in Friend and Stranger and in Friend and Stranger than in Unpleasant condition. Additionally, the linear effect of block order (regardless of task) on the degree of DMN dominance was tested. Both the linear increase and linear decrease of DMN dominance over time yielded no significant results.

**Table 1:** Significant functional connectivity effects.

Contrast	Effect	Area	*x, y, z^1^*	*K*^2^	*p*-FWE^3^
M > F > S > U; DMN > TPN	+	MFG	−30, 30, 42	358	0.002
	+	PCC	2, #x02212;16, 30	254	0.012
	+	Precuneus	−4, −76, 42	489	<0.001
M > F > S > U; DMN	+	MFG	−22, 32, 38	152	0.048
M > F > S > U; TPN	−	AG	−50, −60, 38	480	<0.001
	−	Precuneus	−10, −52, 36	266	0.014
	−	MFG	−40, 16, 44	246	0.019
	−	PCC	6, −22, 28	240	0.022
	−	IFG	−38, 22, −8	221	0.031

**M*, Me; *F*, Friend; *S*, Stranger; *E*, Unpleasant; *AG*, angular gyrus; *IFG*, inferior frontal gyrus; *MFG*, middle frontal gyrus; *PCC*, posterior cingulate. ^1^MNI coordinates of cluster center. ^2^Number of voxels. ^3^FWE-corrected cluster-level *p*-value*.

Next, the Me > Friend > Stranger > Unpleasant contrast was tested for DMN and TPN separately. For DMN, a positive effect was found in the left MFG. For TPN, negative effects were observed in five clusters centered in the left angular gyrus, precuneus, MFG, cingulate gyrus, and inferior frontal gyrus (Table 1). In both cases, the opposite effects were not significant.

## Discussion

In our previous article, the same data have been analyzed with regard to the effect of cultural values on DMN connectivity during self- and other-referential processing. Due to the quite different research question and analytical approach, these results could not be compared with the results of this study, where we tested the hypothesis that DMN-TPN balance during trait adjective judgment task depends on the degree of closeness of the evaluated person to the self. According to this hypothesis, the more the evaluated person is perceived as distant from the self, the more the balance shifts from the DMN to the TPN. We proceeded from the assumption that the degree of subjectively experienced similarity of a person to the self might indirectly be evaluated based on the degree of approval/disapproval of this person. In the instructions that were given to participants, they were asked to choose the best friend, a neutral, and an unpleasant person. Subsequent ratings confirmed that the chosen persons significantly differed on the emotional valence attributed to them. Both in explicit ratings and in the trait adjective judgment task, Friend received the highest and Unpleasant person received the lowest score. The analysis of BOLD activation showed that the linear relationship between the degree of self-closeness and brain activation was significant in the posterior DMN hub, which was most active in the Me and least active in the Unpleasant person condition. Interestingly, the main effect of adjective valence on BOLD activation was also revealed in brain areas overlapping with the posterior DMN hub (see Figure 2A). These areas showed highest activity in response to positive adjectives and lowest activity in response to negative adjectives. This is in line with the behavioral results showing that positive descriptions were most frequently endorsed overall. The interaction between target and adjective valence factors, which was revealed in the left and right sensorimotor cortical areas, clearly shows that on average subjects tend to respond “Yes” (activation of the left sensorimotor area; Figure 3A) when they are presented with positive descriptions in the Me, Friend, and Stranger conditions, or with negative descriptions in the Unpleasant person condition, and to respond “No” (activation of the right sensorimotor area; Figure 3B) in the opposite cases.

Connectivity analysis also confirmed our main hypothesis showing that in a set of brain regions the DMN-TPN difference was highest in the Me and lowest in the Unpleasant person condition. Two out of three significant clusters were situated within the CMS, which have been proposed as the system underlying the human self (Northoff and Bermpohl, 2004; Qin and Northoff, 2011), and one cluster was situated in the left DLPFC, which is also known as an area consistently activated in self- vs. other-referential tasks (Northoff et al., 2006; Morin and Michaud, 2007; Vanderwal et al., 2008). It is interesting that when the linear contrast (Me > Friend > Stranger > Unpleasant) was tested for DMN and TPN separately, significant effects included brain regions belonging to the opposite network. Thus, the Me > Friend > Stranger > Unpleasant contrast was positively associated with the strength of connections between the DMN and the left DLPFC (a CEN region) and was negatively associated with connectivity between the TPN and some DMN regions (angular gyrus, precuneus, PCC). Functional connectivity measures do not allow to reveal the direction of information flow between cortical areas. The use of effective connectivity measures (e.g., dynamic causal modeling, DCM) in a future research may help to elucidate the nature of these interactions. However, the DCM framework only allows to analyze effective connectivity between *a priori* defined ROIs. It does not allow to reveal *a priori* unknown brain regions, which are stronger connected to DMN than to TPN and vice versa, which was our aim in this study.

The theme of DMN-TPN relationship has most thoroughly been discussed in the context of psychopathological disturbances (Hamilton et al., 2011, 2013; Menon, 2011, 2018). The triple network model has been suggested, which posits that a disbalance in the relationships between the DMN, the CEN, and the SAL underlies a wide range of psychopathologies including depression, autism, and schizophrenia (Menon, 2011, 2018). A dominance of the DMN over the TPN has been demonstrated in depression both in clinical and nonclinical samples (Hamilton et al., 2011, 2013; Knyazev et al., 2016, 2018). This dominance correlated with the severity of self-focused rumination (Hamilton et al., 2011), which is a consequence of inherent in depression enhanced self-focus (Watkins and Teasdale, 2004; Grimm et al., 2009). These findings are in line with observations showing that in non-clinical populations, mind wandering, which has been associated with DMN activity (Fox et al., 2015), is related to lower levels of happiness (Killingsworth and Gilbert, 2010). On the other hand, engaging in a demanding activity gives rise to the experience of “flow,” which is accompanied by deactivation of DMN and activation of TPN regions and a positive experience of pleasantness and intrinsic motivation (Csikszentmihalyi, 2000; Ulrich et al., 2016). Meditation-related changes in DMN-TPN balance are accompanied by metacognitive capacities of decentering and self-transcendence (Kang, 2019; King and Fresco, 2019), which essentially mean diminished self-focus and increased attention to the environment. This evidence allows to interpret our results in such a way that the DMN-TPN balance in the trait adjective judgment task reflects the balance between seeing the evaluated person as a part of selfhood vs. an external object. To some degree, this balance may reflect the distinction between a “subjective” (i.e., through the prism of self) and an “objective” point of view or between first- and third-person perspective taking (Northoff and Heinzel, 2006).

An alternative interpretation could be that the observed effects actually reflect differences in emotional valence attributed to different targets. Indeed, all three networks are in different ways associated with emotion processing. The DMN hubs have been linked to social and affective cognition and are anatomically connected to regions involved in emotion generation (Gusnard et al., 2001; Raichle et al., 2001; Sambataro et al., 2014). Moreover, the DMN is active when people experience complex emotions about others’ psychological qualities (Immordino-Yang et al., 2009). The CEN is involved in executive control functions including emotion regulation (Wager et al., 2008; Dailey et al., 2018; Pan et al., 2018), and the SAL also plays essential role in emotional processing (Cauda et al., 2011; Pan et al., 2018). Although, as has been discussed in the “Introduction,” section it is difficult to disentangle the effect of target’s closeness to the self and the effect of emotional valence attributed to the target, some results do not fit this interpretation. In particular, the DMN-TPN balance is higher in the Me than in the Friend condition, despite the fact that emotional valence attributed to the target is higher in the latter case.

Another potential confound, which might have influenced the results is the alleged anticorrelation between the DMN and the TPN. If this anticorrelation is not an artifact of method, as recent findings suggest (Allen et al., 2012; Chai et al., 2012), it should be functionally relevant. Indeed, much evidence shows that the strength of this anticorrelation changes across development (Barber et al., 2013; Knyazev et al., 2017) and in psychopathological conditions (Chai et al., 2011; Hamilton et al., 2011; Marchetti et al., 2012; Knyazev et al., 2016). Moreover, in some cognitive tasks, this anticorrelation might disappear and be replaced with cooperation (Beaty et al., 2016). Some recent findings imply that a dynamic inhibitory control from the CEN to the DMN may partly underlie this anticorrelation (Chen et al., 2013). In any case, if anticorrelation has indeed influenced our findings, this only means that the strength of this anticorrelation during the trait adjective judgment task depends on the degree of closeness of evaluated person to the self.

An important question pertinent to the interpretation of results is to what extent could differences in brain activity/connectivity represent more general differences in task difficulty rather than differences in self-relatedness (or internal/external focus) *per se*? It would make sense that assessing adjectives for less familiar people (i.e., Stranger and Unpleasant condition) would be more difficult than for themselves and close others. It has been shown for instance that the task difficulty might affect whether or not brain activities within MPFC would be dissociated between self- and other-referential processing (Yaoi et al., 2013). RT is frequently used as a proxy for task difficulty. We therefore performed our analyses controlling for RT. In any case, some results are difficult to explain in terms of task difficulty. Thus, mean RT was higher in Me than in Friend condition, implying a greater difficulty in the former than in the latter case. This seems reasonable, because self-evaluation needs an ability to look at the self from a third person perspective and should be more difficult than evaluation of a well-known close friend. Nevertheless, both BOLD activation and connectivity analyses showed that the DMN dominance was higher in the former case. Moreover, it has been shown that task difficulty specifically affects MPFC activity (Huijbers et al., 2010; Yaoi et al., 2013), whereas in our study, activation differences were found in the PCC/precuneus.

Some limitations of the study should be discussed. Our experimental design somewhat differed from the majority of other studies using the trait adjective judgment task, which could be considered a limitation. In many studies, a control condition was included (e.g., whether the adjective is written in upper or lower case) and the person of distant other was frequently modeled by a known political figure (e.g., US president; e.g., Kelley et al., 2002). We have not included these tasks deliberately, because we were interested in comparing conditions that differ on self-relatedness but are equal in all other respects. The upper/lower case and similar tasks are useful as a control condition in the study of a variety of semantic tasks, but, in general, they are too easy to control properly all but self-relatedness aspects of the trait adjective judgment task. Using a known political figure to represent the distant other would be disadvantageous in our case, because the perception of such a figure is frequently associated with emotional valence, which could be different in different participants, varying from love to hate and thus muddying the distinction between close and distant other. Another potential limitation is that we used the same list of adjectives for all targets. The fourfold presentation of the same list of adjectives may result in a kind of learning, i.e., in subsequent occasions, the subject may better understand the meaning of each word and use it more confidently than upon the first encountering of this word. To reduce the effect of surprise upon the first presentation, we used the training session outside the scanner, which allowed the subjects to get familiar with the words. Moreover, the order of blocks (i.e., Me, Friend, Stranger, and Unpleasant) and the order of adjectives within each block were picked out randomly for each subject in order to prevent the confounding effect of learning. If, on the other hand, we had opted for using different lists of words for different targets, this would result in an even more severe problem, because the results of evaluation of different targets would not be strictly comparable. Indeed, it is almost impossible to find for each adjective three synonymous words, which would not have some semantic differences. We additionally tested the influence of block order on behavioral and connectivity measures and found no significant effects. We therefore could be reasonably confident that the observed effects are not confounded by the repeated presentation of the same adjectives. One additional limitation, which follows from the fact that we used the same list of adjectives for all targets, is the impossibility to perform a recognition task after the judgment. Usually, words encoded in the self-condition are better recognized than words encoded in other conditions, the so-called self-referential effect in memory (e.g., Klein and Nelson, 2014). However, in this study, we aimed to compare the processing of different targets, rather than to reveal the unique effect of self-reference.

Summing up, this is the first study that investigated the association between DMN-TPN balance and the degree of self-relatedness in the trait adjective judgment task using fMRI functional connectivity data. We proceeded from an assumption that self- and close-other-processing would engage the DMN more than the TPN, whereas distant-other-processing would engage the TPN more than the DMN. Four conditions (Me, Friend, Stranger, and Unpleasant) were modeled that varied in the degree of self-relatedness and a positive association between the degree of self-relatedness and the degree of DMN dominance was revealed. This dominance was found in the posterior CMS regions and in the left DLPFC. These results show that the association between DMN-TPN balance and self- vs. other-processing focus shows up not only in psychopathological conditions or in meditational practices but is also present in mainstream population in ordinary psychological processes related to social cognition.

## Data Availability Statement

The raw data supporting the conclusions of this article will be made available by the authors, without undue reservation.

## Ethics Statement

The studies involving human participants were reviewed and approved by the Institute of Physiology and Basic Medicine ethical committee. The patients/participants provided their written informed consent to participate in this study.

## Author Contributions

GK planned the study, performed statistical analyses, and wrote the initial draft of the manuscript. AS, AB, EL, and PR participated in data collection. AS and AB designed the experiment. EL and PR wrote programs for running the experiment. All authors contributed to the article and approved the submitted version.

## Footnotes

^1^https://www.nitrc.org/projects/conn

## Abbreviations

ACC: anterior cingulate cortex
BOLD: blood oxygen level dependent
CMS: cortical midline structures
DLPFC: dorsolateral prefrontal cortex
DMN: default mode network
FDR: false-discovery rate
fMRI: functional magnetic resonance imaging
CEN: central executive network
FWE: family-wise error
GLM: general linear model
HRF: hemodynamic response function
ICN: intrinsic connectivity networks
MFG: middle frontal gyrus
MNI: Montreal Neurological Institute
MPFC: medial prefrontal cortex
PCC: posterior cingulate cortex
ROI: region of interest
RT: reaction time
SAL: salience network
TNN: task-negative network
TPJ: temporoparietal junction
TPN: task-positive network.
